# 

**Published:** 1843-11-11

**Authors:** 


### CONTENTS.

A Course of Lectures on the Diseases of Females. By M. Huguier. Lecture 1, - 253

On the So-called "Wood Naphtha" of Dr. Hastings. By William Procter, Jr., - 255

A case of Anomalous Hysteria. By Robert H. Cummins, M. D., of Wheeling, Va., 256

*Clinical Lectures and Reports.*

CaseI.—Tubercular Peritonitis—Arachnitis —Pulmonic Tubercles—Pleuritis—Acute Peritonitis—Death—Autopsy - - 257

Case II.—Tubercular Peritonitis—Phthisis —Arachnitis—Pleuritis—Acute Peritonitis—Hypertrophy of Left Ventricle—Death—Autopsy, - - - - 258

Remarks on Tubercular Peritonitis. By Meredith Clymer, M. D. - - - 258

*Bibliographical Notices.*

On the Effects of Secluded and Gloomy Imprisonment on individuals of the African variety of Mankind, in the production of disease. By Benjamin H. Coates, M. D. 259

The Principles and Practice of Medicine. By John Elliotson, M. D., &c., - - 260

The Anatomy, Physiology, Pathology, and Treatment of Cancer. By Walter Hayle Walshe, M. D., &c., - - - - - 261

Pulmonary Consumption Successfully treated with Naphtha. By John Hastings, M. D., &c., - - - - - 262

*Retrospect of the Medical Sciences.*

Gangrene of the Lung, &c., - - - - 262

Mortality after Amputation, - - - 263

Dr. Simon on Urine in Morbus Brightii, - 263

Influence of the Seasons, - - - - 263

Dr. Simon on Urine in Diseases of the Spinal Cord, - - - - - 264

